# Diplomatic Assistance: Can Helminth-Modulated Macrophages Act as Treatment for Inflammatory Disease?

**DOI:** 10.1371/journal.ppat.1005480

**Published:** 2016-04-21

**Authors:** Svenja Steinfelder, Noëlle Louise O’Regan, Susanne Hartmann

**Affiliations:** Department of Veterinary Medicine, Institute of Immunology, Freie Universität Berlin, Berlin, Germany; University of Pennsylvania, UNITED STATES

## Abstract

Helminths have evolved numerous pathways to prevent their expulsion or elimination from the host to ensure long-term survival. During infection, they target numerous host cells, including macrophages, to induce an alternatively activated phenotype, which aids elimination of infection, tissue repair, and wound healing. Multiple animal-based studies have demonstrated a significant reduction or complete reversal of disease by helminth infection, treatment with helminth products, or helminth-modulated macrophages in models of allergy, autoimmunity, and sepsis. Experimental studies of macrophage and helminth therapies are being translated into clinical benefits for patients undergoing transplantation and those with multiple sclerosis. Thus, helminths or helminth-modulated macrophages present great possibilities as therapeutic applications for inflammatory diseases in humans. Macrophage-based helminth therapies and the underlying mechanisms of their therapeutic or curative effects represent an under-researched area with the potential to open new avenues of treatment. This review explores the application of helminth-modulated macrophages as a new therapy for inflammatory diseases.

## Introduction

**Fig 1:**
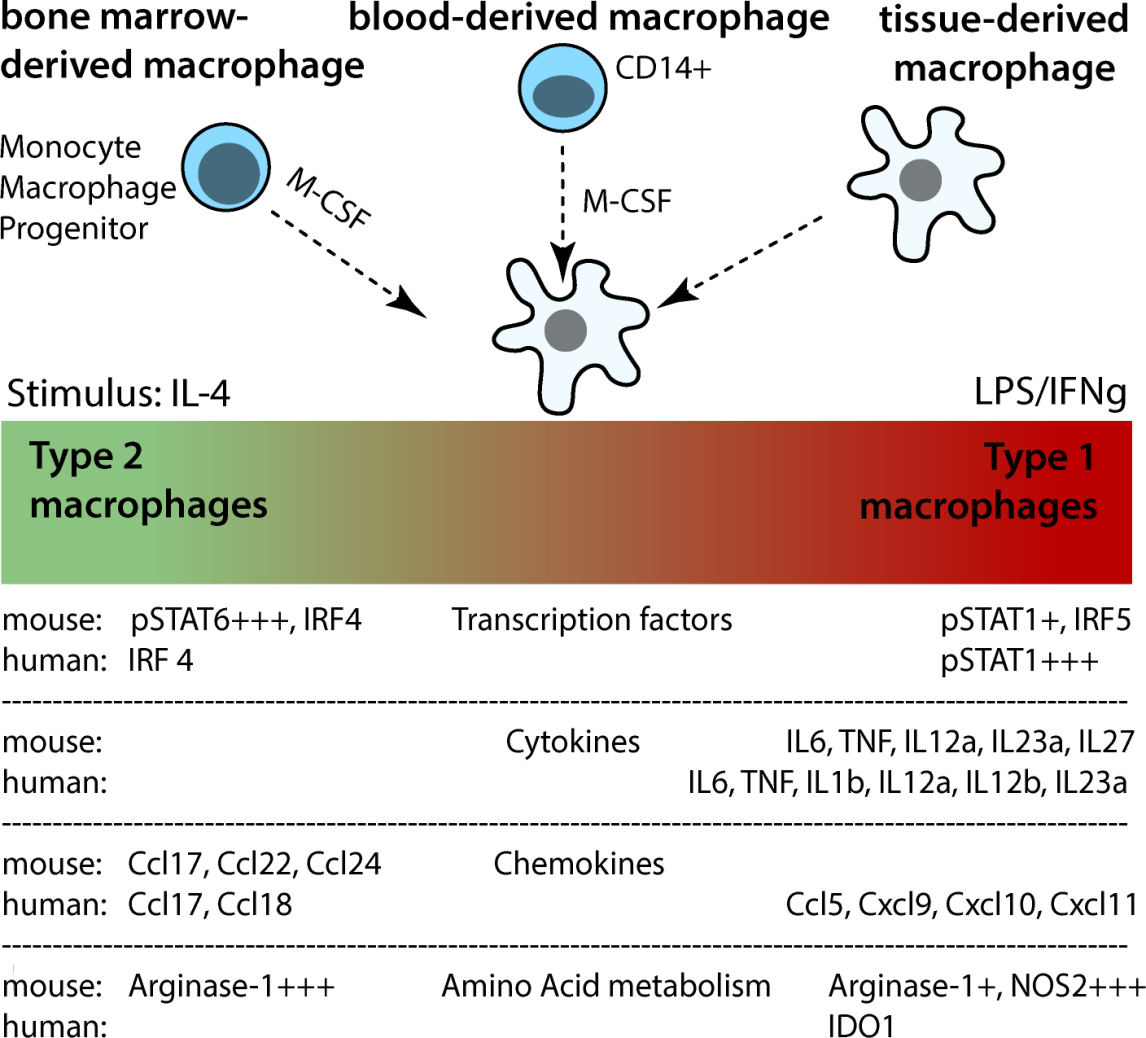
Origin and activation spectrum of murine and human macrophages. Modified from Murray et al. [1] and focused on M1 and M2 macrophages only.

Regulation of macrophage activity and function is essential to balance tissue homeostasis, driving or resolving inflammation in most disease processes. The inflammatory or anti-inflammatory activities of macrophages are shaped in a tissue- and signal-specific manner, enabling macrophages to induce various activation patterns and develop specific functional programs (Fig 1) [1,2].

A recent study in airway hyperreactivity has demonstrated that local macrophages acquire an alternatively activated phenotype (AAM) with regulatory aspects that prevent the development of pathology by inducing antigen-specific CD4^+^ FoxP3^+^ T regulatory (Treg) cells [3]. In a skin allergy model, monocytes that are recruited to the site of inflammation express high levels of the typical AAM markers arginase-1 (arg-1), chitinase-like proteins (CLP), and programmed death-ligand (PD-L)2 and reduce inflammation [4]. Hence, the anti-inflammatory and immunoregulatory functions of macrophages could be harnessed for inflammatory disorders, implying that studies to understand their maintenance and stability in vivo are essential.

Helminths typically induce T helper (Th)2 responses but have also developed multiple ways to regulate the host immune system to ensure their long-term survival in the host. This regulation can affect bystander allergic or autoimmune diseases, and it has become clear that the presence or absence of helminths in humans has a major influence on the prevalence of such diseases. According to the hygiene hypothesis, improvements in public health have reduced incidences of bacterial, viral, and parasitic diseases, which correlate with an increase in chronic autoimmune inflammatory and allergic disorders. Epidemiological studies demonstrate the inverse relationship between helminth infections and inflammatory bowel disease (IBD) [5] or allergies [6,7]. Multiple experimental studies in mice recapitulate this negative correlation and show disease improvement with concurrent helminth infections, allowing underlying mechanisms to be unravelled.

Several immune cells become activated in helminth infection, with Tregs, regulatory B (Breg) cells, and AAM representing master regulators of pathology [8]. This review focuses on helminth-induced immunoregulatory macrophages, which can protect against unrelated inflammation and parasite-induced tissue damage [8–10].

Abundant evidence demonstrates the potential of immunosuppressive, macrophage-targeted therapies in the treatment of renal disease, diabetes, inflammatory diseases, and transplantation rejection. In a chronic inflammatory renal disease model, macrophages polarized in vitro with interleukin (IL)-4 and IL-13 ameliorate disease severity and injury after transfer into mice with the disease [11]. In diabetic mice, transfer of macrophages treated with a combination of IL-4, IL-10, and transforming growth factor (TGF)-β protects up to 80% from the condition [12]. M2 macrophages reduce proinflammatory Th1 and Th17 responses and disease severity in mice with experimental autoimmune encephalomyelitis (EAE), a model of multiple sclerosis (MS) [13]. Similarly, M2 macrophages can protect from septic shock in a model of cecal ligation and puncture [14]. These studies show great promise for the application of macrophages in chronic diseases.

## Helminth-Modulated Macrophages

Macrophages are key innate immune cells that encounter helminths upon initial infection. The macrophage immunoregulatory phenotypes that develop during helminth infection divert anti-helminth immunity to induce host tolerance, parasite survival, and repair of any tissue injury caused by larvae or eggs [15,16].

Murine macrophages that develop in helminth infections express arg-1, resistin-like molecule (RELM)-α, CLPs, mannose receptor C type (MRC)-1 [17], and proliferate in situ [18–20]. In murine models of *Schistosoma mansoni* infection, arg-1–positive macrophages suppress IL-12 and IL-23 production [21]. In *Nippostrongylus brasiliensis* infection, gut macrophages express arg-1, RELM-α, and Ym1 in an IL-4– and IL-13–dependent manner [22], and their depletion allows parasite persistence. Interestingly, neutrophils can also promote the development of M2 macrophages, which subsequently adhere to helminth larvae, increasing their mortality; these macrophages can transfer protection to naïve animals [23]. In human filarial infections, different monocyte phenotypes exist depending on the individual’s disease status. In asymptomatic *Brugia malayi* infection, monocytes express typical M2 markers, which can be recapitulated by stimulation of human monocytes with filarial antigen or live microfilariae in vitro [24–26].

**Fig 2:**
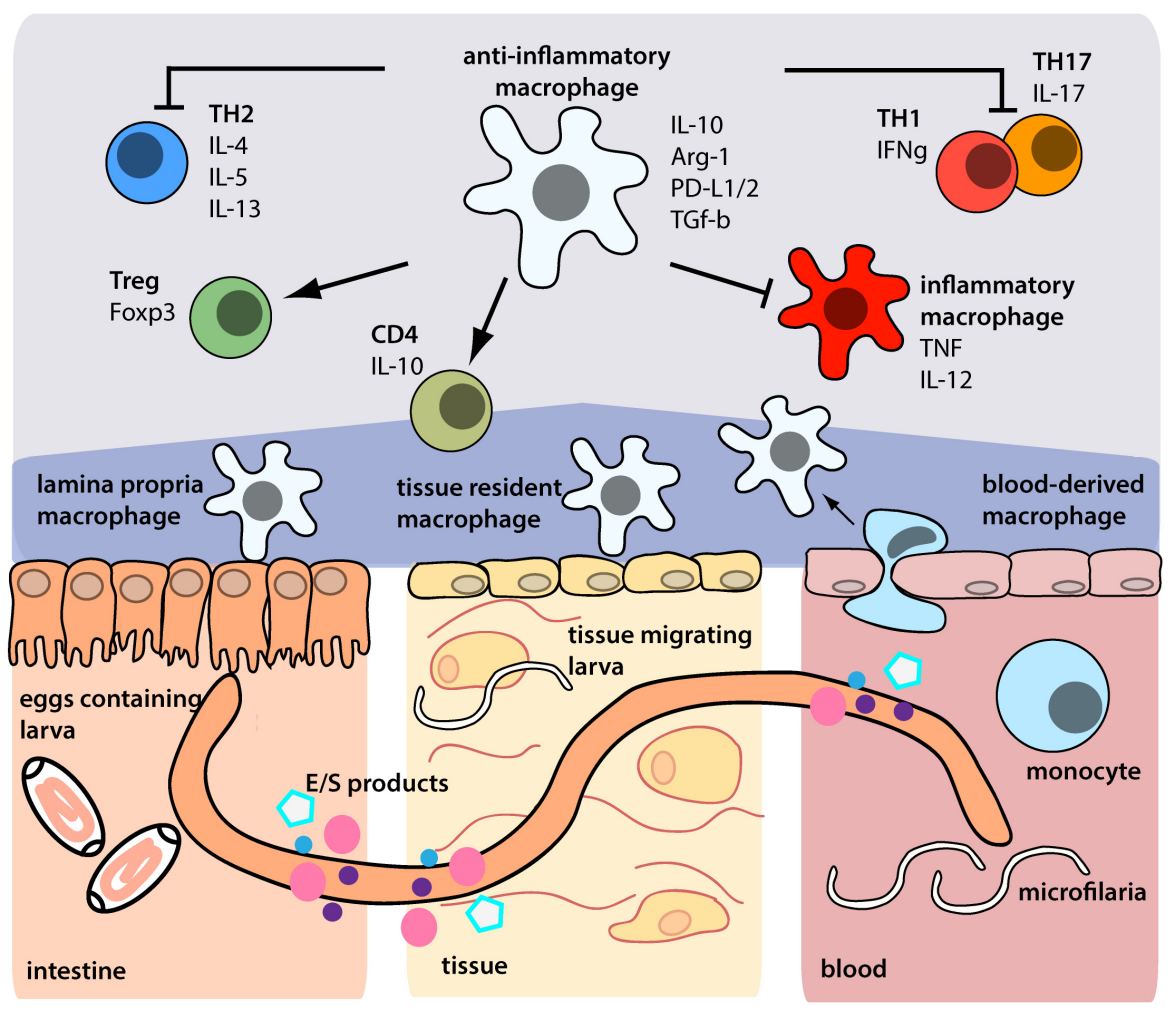
Anti-inflammatory macrophages derived from the intestine, tissue, or blood, stimulated by helminths or their products, and their mechanism of action.

Defined helminth products can also act on macrophages to induce specific regulatory phenotypes; great efforts have been made to identify helminth products with therapeutic potential [27]. A clear example of this is the filarial molecule ES-62 from *Acanthocheilonema viteae*, which targets macrophages to repress IL-12 in cells exposed to lipopolysaccharide (LPS) and interferon (IFN)-γ [28,29]. A cysteine protease inhibitor from *A*. *viteae* (AvCystatin) is recognised and taken up by macrophages to induce phosphorylation of the mitogen-activated protein kinase signalling pathways ERK1/2 and p38, resulting in IL-10 production [30]. These macrophages also express arg-1, PD-L1, and PD-L2, promote IL-10 production in CD4^+^ T cells in a cell contact–dependent manner, and protect against allergy and colitis upon adoptive transfer [9]. In summary, helminths modulate macrophages to develop distinct phenotypes and functions that reduce or prevent host immunopathology by inducing regulatory cell populations or diverting proinflammatory effector cells (Fig 2).

This cell population may be taken advantage of to develop new therapeutic agents and treat unrelated inflammatory diseases (Box 1).

**Box 1. Characteristics of Selected Inflammatory Diseases and Widely Used Animal Models**

**Allergy:** Strong Th2 responses in mucosal tissues or skin to environmental and food antigens involving eosinophils, mast cells, and IgE. Animal model: Allergic airway inflammation using sensitization and challenge with model allergens (ovalbumin).

**Inflammatory Bowel Disease (IBD):** Autoimmune disease. Ulcerative colitis is characterized by a dominant CD4^+^ Th1 response of the colon.

Crohn‘s Disease can occur through the entire length of the gastrointestinal tract and is typically associated with an excess of Th2 cytokines. Animal model: spontaneous development of colitis in IL-10–deficient mice or in T and B cell–deficient mice upon transfer of antigen-experienced T cells. Chemical-induced colitis is based on disruption of the intestinal barrier and T cell response against autologous proteins.

**Diabetes:** Type 1 diabetes (T1D) occurs early in life and is immunologically driven, primarily by a strong CD8^+^ T cell response that destroys pancreatic β cells. Type 2 Diabetes (T2D) is associated with lifestyle and nutrition factors.

Animal model: Nonobese diabetic (NOD) mice develop symptoms of T1D spontaneously at about 12 weeks of age.

**Multiple Sclerosis (MS):** Complex demyelinating inflammatory disorder of the central nervous system involving humoral and cellular (Th1 and Th17) immune responses. Animal model: Experimental autoimmune encephalomyelitis (EAE) is induced by injection of myelin-oligodendrocyte glycoprotein and adjuvants and mirrors major aspects of the complex pathophysiology of MS.

**Rheumatoid Arthritis (RA):** Autoimmune disease causing inflammation and destruction of the joints. It is a systemic disease that exhibits extra-articular manifestations as well. Animal model: Collagen-induced arthritis (CIA). Tissue injection of collagen together with complete Freud‘s adjuvants in susceptible mouse strains.

**Sepsis:** A serious medical condition characterized by dysregulated systemic inflammatory responses towards microbial stimuli followed by immunosuppression. Animal model: Bolus injection of Toll-like receptor agonists or cecal ligation and puncture, which mimics the polymicrobial sepsis observed in human disease.

## Application of Helminth-Modulated Macrophages in Autoimmune Diseases

**Fig 3:**
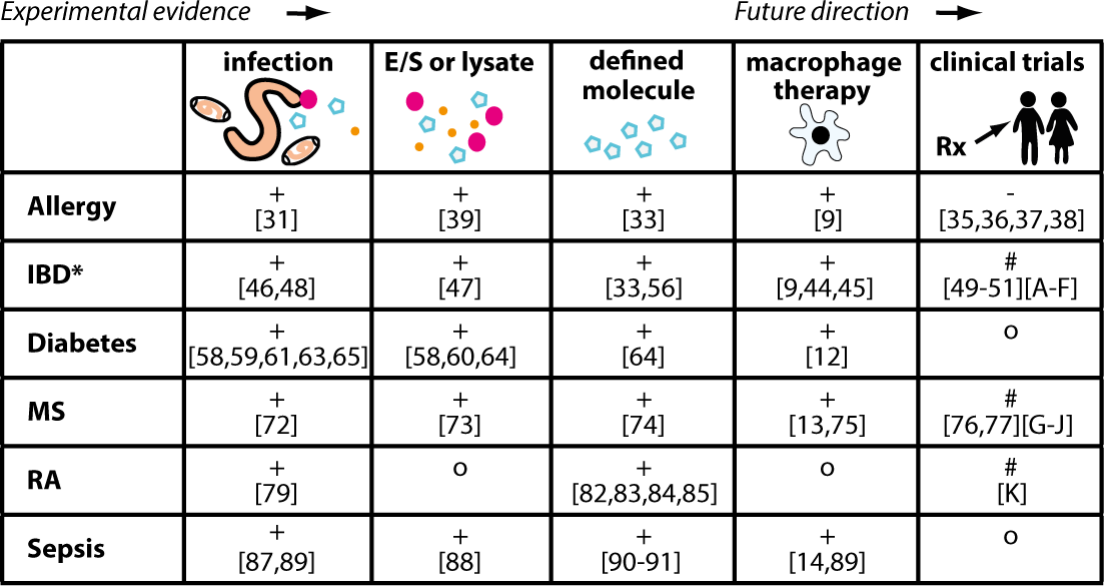
Experimental evidence and clinical trials highlighting the potential of helminth and macrophage therapy. + = positive outcome,— = no improved outcome, # = under investigation, o = no data available, * and coeliac disease. IBD: Inflammatory bowel disease, MS: Multiple sclerosis, RA: Rheumatoid arthritis. [A]: NCT01279577, [B]: NCT01576471, [C]: NCT01434693, [D]: NCT01953354, [E]: NCT01433471, [F]: NCT01661933, [G]: NCT014113243, [H]: NCT00645749, [I]: NCT01006941, [J]: NCT01470521, [K]: DHRS0005323.

It is important to establish whether, once differentiated, the regulatory phenotype of helminth-modulated macrophages is stable enough to treat chronic diseases. We aim to instigate a discussion by reviewing current data on these macrophages in the treatment of inflammatory diseases (Fig 3).

### Helminths and macrophages in allergy and asthma

Allergies are driven by dysregulated Th2 responses, predicting that helminth infection might exacerbate these inflammatory disorders. Nevertheless, the strong regulatory mechanisms employed by helminths suppress Th1- and Th2-mediated diseases. We have previously reviewed helminth infections that mediate protection in allergy-related experimental animal models [31]; we describe here those that illustrate macrophages as potential therapeutic targets for these and other diseases.

Lung macrophages are key players in asthma and develop a defined activation status that modulates adaptive immune responses by local T cells. Despite the fact that lung macrophages are involved in fibrogenesis in asthma [32], it has been shown that tissue-resident macrophages can induce FoxP3^+^ Treg cells [3]. In a murine model of ovalbumin (OVA)-induced airway hyperreactivity, treatment with AvCystatin reduced eosinophil lung recruitment and production of OVA-specific immunoglobulin (Ig)E, total IgE, and allergen-specific IL-4, thereby diminishing disease symptoms. Depleting IL-10 or macrophages reversed these antiallergic effects, implicating the therapeutic potential of macrophages in this model [33,34]. In fact, transfer of AvCystatin-treated macrophages to mice with airway hyperreactivity suppressed clinical disease symptoms [9].

A recent clinical trial focused on helminth therapy in rhinitis [35–37], in which patients were treated with *Trichuris suis* ova. While an antiparasitic immune response developed in these patients, neither a redirection of allergen-specific immune responses nor a therapeutic effect was achieved. Similarly, experimental hookworm infection did not lead to improved outcomes in a clinical trial with patients suffering from asthma [38]. However, allergic mice treated with excretory/secretory (E/S) products from *T*. *suis* had reduced allergic airway hyperreactivity after challenge [39], which might be a reflection of the route of application or the amount of helminth-derived immunomodulatory molecules available in this setting. Thus, promising preclinical data need to be translated to show definitive clinical benefits for patients with allergic disorders.

### Helminths and macrophages in inflammatory bowel disease and coeliac disease

Distortion of the intestinal barrier and immune response to intestinal bacteria can lead to IBD, including ulcerative colitis and Crohn's disease. Tissue macrophages are present in high numbers in the intestine and present a good target for helminth therapy because of their multiple activation states. In healthy individuals, lamina propria macrophages maintain intestinal homeostasis by inducing Tregs [40,41], while in active IBD, macrophages contribute to pathology by expressing multiple proinflammatory cytokines [42]. M1 macrophages invading the intestinal tissue drive the disruption of the epithelial barrier through dysregulation of tight junction proteins and epithelial apoptosis [43]. In contrast, patients with inactive Crohn's disease have higher levels of M2 macrophages [44], which are also important in inducing protection against IBD in mice [45]. Thus, helminth-induced M2 macrophages and Tregs may contribute to protection against IBD. Mice infected with *Hymenolepis diminuta* [46] and treated with the adult worm extract [47] or treated with IL-4/IL-13–differentiated M2 macrophages [44] have significantly reduced pathology in experimentally induced colitis; this protective effect is abrogated when IL-10 [46] or macrophages [44] are depleted. Murine infection with *S*. *mansoni* prevents colitis in a macrophage-dependent but IL-4- and IL-13–independent manner, representing another population of suppressive macrophages [48].

Intriguingly, experimental hookworm infection combined with gluten microchallenge induces tolerance in patients with coeliac disease, an autoimmune disease resulting from gluten intolerance [49]. The contribution of macrophages was not evaluated in this setting.

Multiple clinical trials are investigating the use of *T*. *suis* ova therapy in IBD and have shown moderate success (see Fig 3) [50,51]. The safety of this treatment, threatened by the colonization and invasion of the host by *T*. *suis*, has been much debated and requires treated patients to be monitored closely [52–55]. An alternative approach would be to administer characterized helminth products such as AvCystatin or transgenic probiotic bacteria expressing helminth immunomodulators, which lead to diminished disease scores in murine IBD models by reducing numbers of inflammatory macrophages [33,56]. Nevertheless, there are currently no clinical trials addressing the role of helminth-modulated macrophages in protection against IBD. Future studies should translate the encouraging experimental evidence into clinical benefits for patients.

### Helminths and macrophages in diabetes

Both environmental and genetic factors play a role in the development of diabetes, and incidences of this condition have increased dramatically in the past 30 years in developed and newly industrialized countries [57]. Studies have demonstrated an inverse correlation between diabetes (type 1 [T1D] and type 2 [T2D]) and helminth infections [58]. Helminth products have also been demonstrated to reduce incidences of diabetes in animal models [58–61]. Early studies suggested that macrophages could exacerbate T1D, in which macrophage depletion ameliorated disease [62]. As T1D is a Th1-driven disease, it is likely that the macrophages involved are classically activated, which could be redirected by a helminth infection. Nonobese diabetic (NOD) mice infected with *Heligmosomoides polygyrus* have augmented numbers of Tregs and Th2 responses as well as an infiltration of M2 macrophages and increased IL-10 expression in the pancreatic lymph nodes [63]. Injection of schistosome egg antigen into NOD mice induces arg-1 and RELM-α expression in macrophages and modulates T cell responses [64]. In another diabetes model, infection with *Taenia crassiceps* attenuates disease in two different mouse strains and is accompanied by high levels of IL-4 and M2 macrophages [65]. To date, there are no clinical trials examining the application of helminths or macrophages in T1D, indicating an open area for future research.

### Helminths and macrophages in multiple sclerosis

MS is an inflammatory autoimmune disorder driven by dysregulated Th1 and Th17 responses, resulting in a demyelinating disease that affects the central nervous system (CNS). Environmental and genetic factors may be involved in disease onset [66]. As MS progresses, acute inflammatory lesions develop when the integrity of the blood–brain barrier is disturbed, with CD4^+^ Th1, Th17 cells, and CD8^+^ cells becoming activated by mature dendritic cells [67].Various studies have demonstrated that helminth-infected patients with MS have fewer relapses and inflammatory changes than uninfected patients, while removal of helminth infection exacerbates MS disease [68–70].

Different helminth species have been studied for their ability to modulate unwanted inflammatory responses in MS [71]. Mice with EAE immunised with *S*. *mansoni* eggs have lower disease severity; clinical scores and cellular infiltrates are reduced, and CD11b^+^ macrophages isolated from the CNS show decreased IL-12 expression [72]. Schistosomal egg antigen and a single schistosome glycan were also effective in protecting mice against EAE [73,74]. The importance of M2 macrophages that produce IL-10 and protect mice from developing EAE has also been described [75].

While no clinical trials currently exist that use macrophages to treat MS, trials using *T*. *suis* ova (TSO) or hookworm larvae are underway or already present results from a small cohort of patients (Fig 3). While both studies show that TSO is safe, the therapeutic effect is ambiguous: one study reports a decrease in the number of CNS lesions observed by magnetic resonance imaging [76] while a comparable study did not detect clinical improvement [77].

### Helminths and macrophages in rheumatoid arthritis

Multiple experimental helminth-based treatment strategies have been tested in rheumatoid arthritis (RA), a chronic inflammatory disorder [78]. While the exact disease cause is unknown, dysregulated immune responses are important, as high levels of tumour necrosis factor (TNF) and IL-1β have been detected in inflamed synovial membranes. T cells from synovial tissue express Th1- and Th17-associated cytokines and activate neighbouring macrophages that release large amounts of TNF and IL-1β. These and other macrophage-derived proinflammatory cytokines drive much of the inflammation and implicate macrophages as key players in disease [79]. Current treatments include nonsteroidal anti-inflammatory drugs, which can have potentially detrimental long-term side effects [80]. ES-62 shows great potential to treat dysregulated inflammatory disorders [81]. ES-62 prevents collagen-induced arthritis when injected into mice by downregulating IL-17 and MyD88 [82] and restoring levels of IL-10-producing B cells and reducing intra-articular plasma cell infiltration [83]. Introduction of ES-62 in a coculture of T cells from patients with RA and macrophage cell lines significantly reduced macrophage TNF expression compared with ES-62–untreated cells [84]. A synthetic analogue of ES-62 prevented experimental arthritis and inhibited macrophage-derived IL-1β [85]. Numerous therapies for RA are in preclinical or clinical trials, which aim to neutralise or inhibit many macrophage-related disease-driving mechanisms [86]. However, as yet, only one clinical trial assesses helminth infection as a potential therapy for RA (Fig 3).

### Helminths and macrophages in systemic inflammation

Recently, it was shown that helminths and their products can decrease the prevalence of sepsis and improve the outcome of systemic bacterial infection and inflammation [87–91]. Epidemiological data demonstrated a lower prevalence of filarial infection in patients with sepsis than in healthy individuals, suggesting that preexisting helminth infection prevents sepsis development [87]. Fundamental evidence demonstrating that helminth-modulated macrophages improve sepsis came from a murine experimental filarial infection, in which gene expression profiles of macrophages modulated by *Litosomosoides sigmodontis* illustrated decreased Toll-like receptor (TLR) responsiveness. Transfer of macrophages from *L*. *sigmodontis*–infected mice into naïve recipients improved sepsis outcome in a TLR2-dependent but AAM-independent manner [89]. Macrophages from patients with sepsis expressed reduced sepsis-inducing inflammatory cytokines after treatment with *Trichinella spiralis* E/S products [88]. Similarly, a *T*. *spiralis* cathepsin B–like protein ameliorates intestinal ischemia/reperfusion injury, a model for systemic inflammation, by promoting a switch from M1 to M2 macrophages [91]. Furthermore, a single helminth molecule from *Fasciola hepatica* (fatty acid–binding protein; FABP or Fh12) can suppress serum inflammatory cytokines in a septic shock model. This was accompanied by suppression of proinflammatory cytokines and nitric oxide synthase-2 (NOS2) in macrophages [90] and demonstrates the potential of macrophages in this disease setting.

## Macrophages in Cell Therapy: A Potential Treatment Option

For macrophage-based therapies, one must consider the possibility of phenotype reversion after transfer. The phenotype and function of a particular macrophage subset develops from the combined integration of tissue-specific and environmental cues, such as inflammation or infection, which can lead to epigenetic imprinting [19,92]; however, the stability of the therapeutic macrophage phenotype must be determined.

Murine studies have shown that transferred macrophages can block pathology independently of the perturbed environment they encounter [9,12,13,45,75,89]. One particular macrophage subset can confer protection upon transfer in mice and humans. Murine macrophages stimulated with IFN-γ have significant anti-inflammatory characteristics, mitigating colitis and prolonging allograft survival [93,94]. Human macrophages stimulated with IFN-γ in vitro and administered to patients undergoing renal transplant significantly reduced the required dose of immunosuppressant drugs and improved transplanted kidney function [95]. These macrophages conferred immunosuppression on T cells, which was partly mediated by the indoleamine 2,3-dioxygenase and likely induced nutrient deficiencies in alloreactive T cells [94]. Although the mechanism of action of these macrophages is different to that of helminth-induced macrophages, it exemplifies how these powerful cells can redirect undesired immune responses in disease settings.

The macrophage population that bears sufficient therapeutic function in a given environment must be carefully evaluated. Alongside macrophages, other immune cells are involved in helminth-derived immunomodulation. The application of one helminth-modulated cell population cannot represent the full spectrum of immunomodulation compared with a chronic helminth infection, which can induce changes in microbiota [38,96], mediating a therapeutic effect [97], but it might be enough to reset the diseased environment to homeostasis.

## What Does the Future Hold for Helminth-Based Therapies?

The studies discussed herein demonstrate the potential of helminth infections and, in particular, helminth-induced macrophages to treat inflammatory disorders; in some cases, clinical trials are already underway. However, the mode of application must be addressed to determine the safest and most effective route for patients. Is it best to treat the patient with a patent infection or with isolated stages (e.g., eggs)? Is it best to apply specific helminth-derived products (e.g., AvCystatin, ES-62, *T*. *spiralis* cathepsin B–like protein) or to stimulate in vitro and reinfuse a patient’s own macrophages? Live infections provide a rapid path to clinical trials compared with identifying and characterising defined products. Nevertheless, live infections remain infectious, and can induce pathological consequences in the host, especially in immunocompromised individuals [98]. In contrast, defined products can be produced recombinantly in high quantities at relatively low costs. Defined products allow efficient site-directed and prolonged application, e.g., through the use of carriers like probiotic bacteria that colonize and release the molecules in targeted tissues [56]. Generating transgenic auxotrophic strains that release powerful helminth products will enable the use of such techniques without risking contamination of the environment. However, helminth products themselves may be immunogenic, and thus, a further therapeutic alternative is the synthesis of small-molecule analogues, as described for ES-62. New targets identified by large-scale technologies (proteomics, metabolomics, genomics) combined with bioinformatics aid the discovery of novel pathways and molecules that can translate helminth–or helminth product–derived immunomodulating strategies into efficient therapies [27].

The experimental models that illustrate the prospect of helminth-modulated, macrophage-based therapies provide hope that safe and effective treatments for humans are a viable option. The abilities of macrophages to regulate T and B cell function and cytokine production highlight this innate cell population as a powerful tool in therapy development. However, the stability of transferred macrophages must be established. The fact that clinical trials employing the regulatory effects of helminths or immune-suppressive macrophages are underway is extremely encouraging and indicates that research in this direction should be pursued.
